# Parental food consumption and diet quality and its association with children’s food consumption in families at high risk of type 2 diabetes: the Feel4Diabetes-study

**DOI:** 10.1017/S1368980022002245

**Published:** 2022-12

**Authors:** Lubna Mahmood, Luis A Moreno, Paloma Flores-Barrantes, Christina Mavrogianni, Peter Schwarz, Konstantinos Makrilakis, Stavros Liatis, Greet Cardon, Ruben Willems, Imre Rurik, Sándorné Radó, Tsvetalina Tankova, Violeta Iotova, Natalya Usheva, Yannis Manios, Esther M Gonzalez-Gil

**Affiliations:** 1 Growth, Exercise, Nutrition and Development (GENUD) Research Group, University of Zaragoza, Zaragoza 50009, Spain; 2 Instituto Agroalimentario de Aragón (IA2), Zaragoza, Spain; 3 Instituto de Investigación Sanitaria de Aragón (IIS Aragón), Zaragoza, Spain; 4 Centro de Investigación Biomédica en Red de Fisiopatología de la Obesidad y Nutrición (CIBERObn), Instituto de Salud Carlos III, Madrid, Spain; 5 Department of Nutrition and Dietetics, School of Health Science and Education, Harokopio University, Athens, Greece; 6 Department of Medicine III, Technical University of Dresden, Dresden, Germany; 7 Department of Propaedeutic Internal Medicine, National and Kapodistrian University of Athens, Athens, Greece; 8 Department of Movement and Sports Sciences, Ghent University, Gent, Belgium; 9 Department of Public Health and Primary Care, Ghent University, Gent, Belgium; 10 Faculty of Health Sciences, University of Debrecen, Debrecen, Hungary; 11 Department of Diabetology, Clinical Center of Endocrinology, Medical University of Sofia, Sofia, Bulgaria; 12 Department of Social Medicine and Health Care Organization, Medical University of Varna, Varna, Bulgaria; 13 Institute of Agri-food and Life Sciences, Hellenic Mediterranean University Research Centre, Heraklion, Greece; 14 Department of Biochemistry and Molecular Biology II, Instituto de Nutrición y Tecnología de los Alimentos, Center of Biomedical Research (CIBM), Universidad de Granada, Granada, Spain

**Keywords:** Food consumption, Diet quality, Type 2 diabetes, Parents, Children

## Abstract

**Objective:**

To examine the parental food consumption and diet quality and its associations with children’s consumption in families at high risk for developing type 2 diabetes mellitus across Europe. Also, to compare food frequency consumption among parents and children from high-risk families to the European Dietary guidelines/recommendations.

**Design:**

Cross-sectional study using Feel4diabetes FFQ.

**Setting:**

Families completed FFQ and anthropometric measures were obtained. Linear regression analyses were applied to investigate the relations between parental food consumption and diet quality and their children’s food consumption after consideration of potential confounders.

**Participants:**

2095 European families (74·6 % mothers, 50·9 % girls). The participants included parent and one child, aged 6–8 years.

**Results:**

Parental food consumption was significantly associated with children’s intake from the same food groups among boys and girls. Most parents and children showed under-consumption of healthy foods according to the European Dietary Guidelines. Parental diet quality was positively associated with children’s intake of ‘fruit’ (boys: *β* = 0·233, *P* < 0·001; girls: *β* = 0·134, *P* < 0·05) and ‘vegetables’ (boys: *β* = 0·177, *P* < 0·01; girls: *β* = 0·234, *P* < 0·001) and inversely associated with their ‘snacks’ consumption (boys: *β* = –0·143, *P* < 0·05; girls: *β* = –0·186, *P* < 0·01).

**Conclusion:**

The present study suggests an association between parental food consumption and diet quality and children’s food intake. More in-depth studies and lifestyle interventions that include both parents and children are therefore recommended for future research.

Lifestyle behaviours have their onset during childhood and their development depends on the familial environment, like parental habits and preferences, among others^(1)^. Among the lifestyle behaviours, dietary habits have been specially associated with the development of type 2 diabetes (T2D) in both children^(2)^ and adults^(3)^ and the pre-state condition of T2D and insulin resistance (IR). There is a familial link regarding T2D as there is a genetic component meaning that children from parents with T2D are more likely to have this condition^(4)^. However, there are not so many studies assessing the association between parental IR and children’ IR. Recently, it has been observed that children from parents at risk of T2D have less family meals frequency^(5)^. Also, in a previous study with European data, it has been observed that children from parents at risk of T2D that already had IR were found to have higher odds of unhealthy lifestyle patterns^(6)^. Moreover, having IR and obesity during childhood could increase the risk of developing T2D^(4)^. Therefore, assessing those families that are already at risk of T2D could help to get a better insight of this specific population as children from these families already have worse lifestyle behaviours^(6)^ and less family meals frequency^(5)^.

It has been observed that children’s dietary intake is largely influenced by parental diet and eating behaviours^(7)^. In detail, parents act as role modelling for their children, and they are the ones who shape the home food environment, make food available and accessible to children, influence how a child thinks about food, and, accordingly, start forming their own food preferences and eating behaviour^(8)^. Thus, role modelling behaviours were recommended for parents through providing healthy foods, modelling healthy eating and increasing encouragement to eat healthy foods^(9)^. In this sense, a cross-sectional observational study on 145 parents and their preschool children in Houston found a strong relation between portions offered and served by parents and the amounts that children consumed during a regular meal^(10)^. Moreover, in Japan, results of questionnaire answered by 244 mothers of children aged 3–5 years found that mothers’ preferences, as well as food habits, affected their children’s food intake^(11)^. For instance, a recent cross-sectional study with baseline data from a multicentre European study were collected in 2016 and included 10 038 families from six European countries found that fathers’ intake of fruits and vegetables (FV) was positively associated with children’s daily intake of these foods^(12)^. Similarly, previous studies on family’s eating habits showed that the low FV consumption of parents and their children are strongly related^(13,14)^, suggesting that children consider parents’ food preferences as their models^(15)^, and this process was found to be stronger during early childhood^(16)^. Moreover, according to cross-sectional and cohort studies on the role of parental control practices and home food environment, positive associations have been found between parental healthy food choices and high FV consumption in children^(17,18)^, as well as low intake of unhealthy snacks^(19)^. Likewise, in a recent survey conducted among 104 Italian children, children were found to consume more fish in families where parents used to cook and include more fish in their meals^(20)^.

Diet quality (DQ) is broadly defined as a dietary pattern, frequently used to describe how well an individual’s diet conforms to the key food groups recommended in dietary guidelines^(21)^. High DQ thereby reflects a healthier food intake^(21,22)^ while improvements in DQ have been associated with lower T2D risk^(21)^. However, very few studies have examined the association between parents’ DQ and children’s food intake^(21)^. In general, previous studies using DQ indicators^(23,24)^ showed that improvement in parental DQ was associated with healthier dietary intake of children, like more intake of FV and less consumption of food high in fat and sugar.

To date, the majority of studies looking at parental and children food consumption and DQ presented findings considering only one gender or population with specific race/ethnicity, with healthy subjects or for specific age groups. To our knowledge, no study yet has examined the association between familial dietary habits among populations at high risk of T2D which could give a better insight of the associations in this specific population. Therefore, the aim of the present study was to examine the parental food consumption and DQ and its associations with children’s consumption in families at high risk for developing T2D across Europe. A secondary aim was to compare the food frequency consumption of parents and children from high-risk families according to the European Dietary guidelines/recommendations.

## Methods

### Study design

This cross-sectional study was conducted as a part of the Feel4Diabetes-study, a European interventional study which included a school- and community-based intervention, aiming to promote healthy lifestyle and tackle obesity and obesity-related metabolic risk factors for the prevention of T2D among families from vulnerable groups in six European countries^(25)^. The participating countries were classified as low-income countries (Bulgaria and Hungary), countries under austerity measures (Greece and Spain) and high-income countries (Belgium and Finland). Vulnerable groups were defined as the population in low-/middle-income countries and families from low-socioeconomic neighbourhoods in high-income countries^(6,25)^. In each country, primary schools were randomly selected and recruited in selected provinces with low socioeconomic status areas. All parents having children in the first three grades of primary school were invited to participate. Data were collected at baseline (2016), first (2017) and second year (2018) of the study by well-trained researchers, while the initial recruitments were done between January and November 2016. The current paper used the baseline cross-sectional data only. Feel4Diabetes-study is registered within the clinical trials registry http://clinicaltrials.gov, (NCT02393872), and more details regarding study design can be found elsewhere^(26)^.

### Study sample

**Fig. 1:**
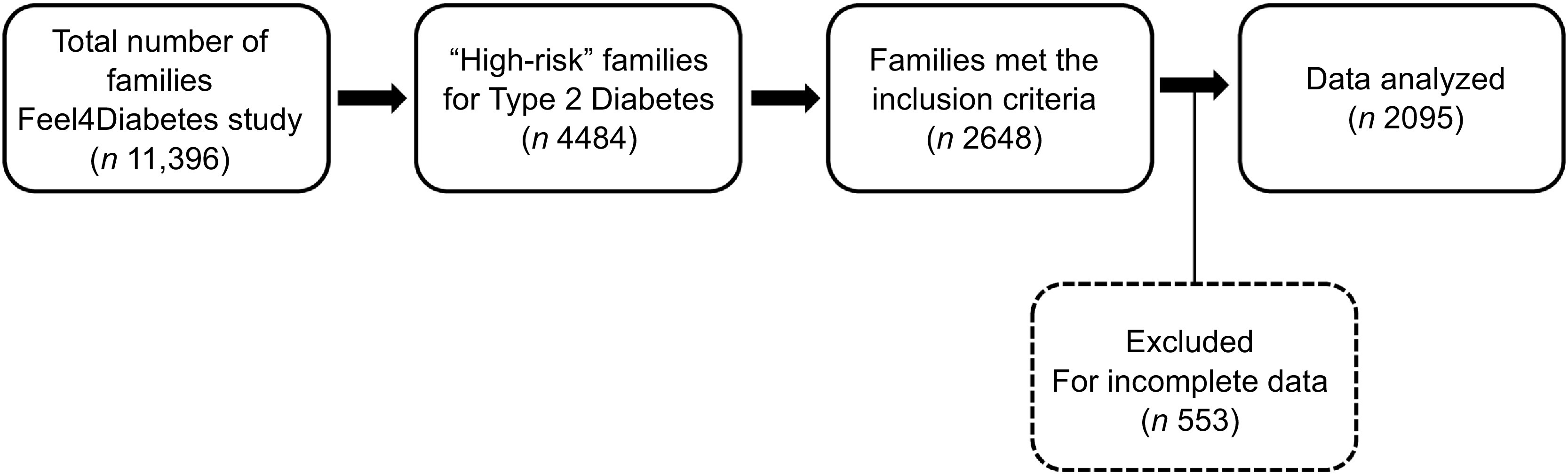
Flow diagram of participants throughout the study

Out of the total sample of 11 396 families, 4484 families were identified as ‘high-risk families’ at baseline. The Finnish Diabetes Risk Score (FINDRISC) questionnaire was used to identify the ‘high-risk’ families based on T2D risk estimation^(6,27)^. It is a reliable and valid questionnaire that consisted of eight questions related to age, blood pressure medication, history of high blood glucose, family history of diabetes, BMI, waist circumference, physical activity and consumption of FV^(27)^. The FINDRISC score ranged from 0 to 26, and a family was considered at ‘high risk’ if at least one parent fulfilled the FINDRISC cut-off point that was set as ≥ 9, indicating an increased risk of T2D^(6,27)^. The specific inclusion criteria were parent with one primary school-aged child (6–8 years old), who completed two questionnaires: FFQ and eating behaviour questionnaires as well as energy balance-related behaviour questionnaires (one for adult and one for children). From 2648 families that met the inclusion criteria (response rate = 59·1 %), 553 were excluded for incomplete information and lack of weight and/or height measurements, and 2095 were included in this study. Flowchart is shown in Fig. 1.

### FFQ

Self-reported questionnaires were filled out by one of the parents, who completed these questionnaires both for him/herself and their child. For the present study, relevant demographic data such as age, sex, parental employment, education and marital status were included. Also, measures on food consumption from parents and children were considered, such as meal frequencies and selected food items consumed. The FFQ were derived from a questionnaire developed for the National T2D prevention program in Finland (FIN-D2D)^(28)^, with some modifications so as to be relevant for the target multi-country population of the Feel4-Diabetes study. The initial forms of the FFQ were developed in English language and then translated back to the language of each participating country and back to English to ensure quality and reliability. The FFQ used is a validated tool, and a reliability study was conducted in 191 pairs of parents and their children. Parents completed the questionnaires on two occasions, within a 1–2-week interval. Reliability was tested by the intra-class correlation coefficients of test–retest^(28)^. The questionnaires were culturally adapted for the target population of the Feel4Diabetes-study across the six countries. The questionnaire for children was similar to that of parents, except for excluding questions regarding coffee and alcohol consumption. The questionnaire included various food groups such as milk and milk products, cereals, fat, fruits, vegetables, legumes, red meat, white meat, fish and seafood, nuts, salty snacks and sweets. The answers were presented as frequency of consumption based on a specified portion size of each food item and options included the following: on a weekly (less than 1, 1–2, 3–4, or 5–6 times per week) or daily basis (1–2, 3–4, 5 times or more per day). In this study, the consumption of each food item was converted to daily intake in grams through multiplying the number of servings consumed by the standard portion size. The food portion sizes provided of both parents and children were similar, also the portions were defined with a household unit and placed under the questions. Whereas, the listed answers provided the frequency of consumption of the specified portion of each food item^(28)^.

### Feel4Diabetes Healthy Diet Score

In the current study, the Healthy Diet Score (HDS) was used to assess the parental DQ as a validated indicator based on Feel4Diabetes-study dietary questions and tested before over families at high risk of T2D^(6,24)^. The DQ was assessed using only adults’ food consumption data as HDS was developed for adults. While, the main components of the HDS were based on a total of twelve Feel4Diabetes intervention goals related to food behaviour and food choices and were obtained from the FFQ of the Feel4Diabetes-study^(6,24)^. These components included family meals, breakfast, whole-grain cereals, salty snacks, sweet snacks, oils and fats, low-fat dairy products, nuts and seeds, red meat, sugary drinks, vegetables, fruits and berries^(24)^. A maximum score of 6 was given to salty snacks, sweet snacks, low-fat dairy, nuts and seeds consumption. A maximum score of 8 was given to the frequency of family meals and the consumption of oils and fats. The rest of the components received a maximum score of 10^(24)^. The total score ranged from 0 to 100, in which higher scores indicate a better diet quality while higher scores of sugary drinks, red meat, salty snacks and sweet snacks indicated lower consumption. More details regarding the scoring of HDS can be found elsewhere^(6,24)^.

### Anthropometric measurements

The height and weight of parents were self-reported, while for children were objectively measured with light clothing and without shoes at schools by a well-trained research team^(26)^. Weight was measured by Seca 813 and recorded to the nearest 0·1 kg, and standing height was measured by Seca 217 and recorded to the nearest 0·1 cm^(26)^. BMI was calculated as weight (kg) divided by height (m) squared. Finally, children’s BMI z-scores were calculated according to Cole *et al.* ^(29)^ to obtain an optimal measure for their weight in accordance with their sex and age.

### Statistical analysis

**Fig. 2:**
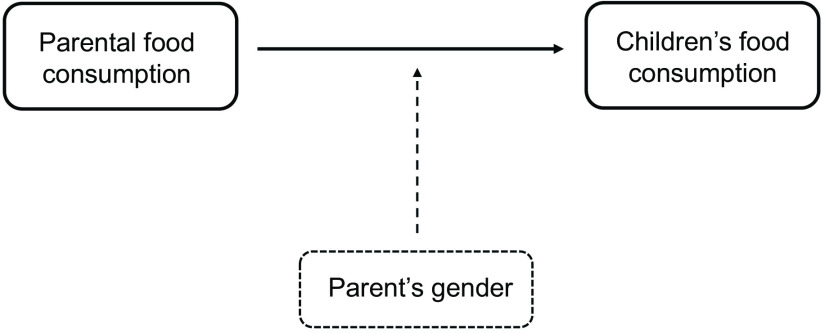
The moderation effect of parental age on the relationship between parents’ and children food consumption

Normality for data was checked using the Kolmogorov–Smirnov test. Descriptive statistics were computed to describe the participant’s characteristics and presented as mean and standard deviation. The frequencies of food consumption of parents and children were presented as percentages (%) and compared to the European Food-Based Dietary Guidelines^(30)^. The HDS for parents was calculated with a total score ranged from 0 to 100, in which higher scores indicating better quality diet. Multiple regression analyses were used to examine the association between parents’ consumption from different food groups and parents’ HDS with the children’s food consumptions by sex. The analyses of children were split by sex as new literature on sex differences in eating behaviours among pre-pubertal children identified sex differences in appetitive traits, food intake, food acceptance, self-regulatory eating and neural response to food images^(31)^. The analyses were adjusted for age, country, educational level, parental sex and BMI of parents and children. Multiple regression analyses were also performed to assess the association between mothers’ consumption from different food groups and mothers’ HDS with the children’s food consumptions by sex. These analyses were adjusted for age, country, educational level and BMI of mothers and children. For regression models, the analysis of residuals confirmed the assumptions of linearity, and the sample size requirement for the sex-specific models was also met. The moderating role of parental gender was tested in the relationship between parents’ and children’s food consumption (Fig. 2). Since the majority of parent’s sample was mother, a sensitivity analysis was conducted to check if the significance of parents’ results was similar in mothers-only sample. Data were analysed with IBM SPSS Statistics for Windows, Version 26.0. IBM Corp, with a *P* < 0·05 representing statistical significance for all tests.

## Results

### Characteristics of study participants

Descriptive statistics of the sample and variables can be found in Table 1. In total, data of 2095 parents and children from high-risk families were analysed (mean age parents: 38·87 ± 5·32 years; 74·6 % females (mothers); mean age children: 7·24 ± 1·0 years; 50·9 % girls). The majority of parents were employed (72·9 %) and around 62 % of them had a tertiary education of more than 13–14 years (e.g. bachelor program).

**Table 1:** Characteristics of the study participants at baseline

Characteristics	Parents			Children		
	%	Mean	sd	%	Mean	sd
Age (in years)		38·87	5·32		7·24	1·0
Sex (% females)	74·6			50·9		
Education level (% high education*)	65·9			–		
Employment status (% employed)	62·3			–		
Marital status (% married)	78·1			–		
Body weight (kg)		78·4	17·4		30·4	7·9
Height (cm)		166·5	8·7		130·8	8·1
BMI (kg/m^2^)†		28·1	5·57		17·62	3·08
BMI z-score	–				0·73	1·10

*n* 2095 parents and children. This table provides mean ± sd for the continuous variables and frequency (%) for the categorical variables.

* 13–14 years of education or more.

† BMI z-scores were calculated according to Cole *et al.* ^(29)^

### Association between dietary intake of parents and children

**Fig. 3:**
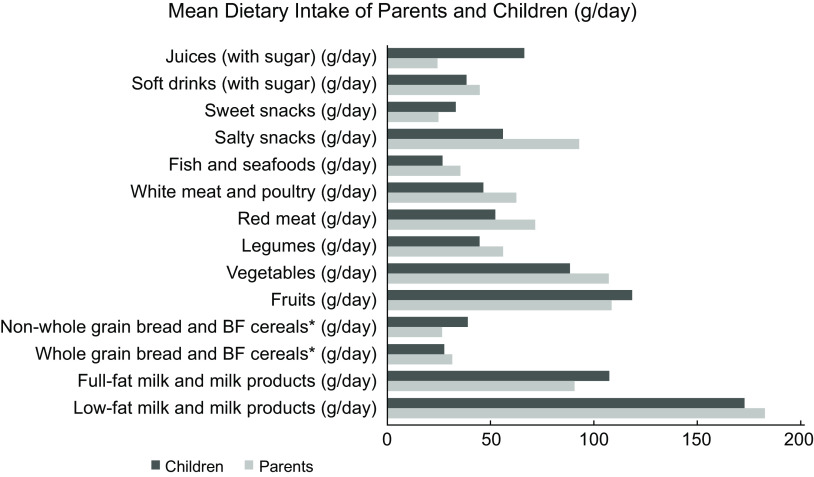
Average dietary intake (g/d) of parents and children from the Feel4Diabetes-study for different food groups and beverages

The mean food intake of parents and children (g/d) is presented in Fig. 3. As shown in Table 2, parental consumption of most food groups was significantly associated with children’s intake among both boys and girls. Parental intake of ‘full-fat milk and milk products’ was not associated with children’s intake from the same group among both boys and girls. Also, parental ‘salty snack’ intake did not show any significant association with boys’ intake from the same food group.

**Table 2:** Association between dietary intake of parents and corresponding intake of the same food groups in their children

	Children’s food consumption			
	Boys		Girls	
Parental dietary intake (food groups)	*β*	95 % CI	*β*	95 % CI
Low-fat milk and milk products†	0·282***	0·091, 0·420	0·341***	0·111, 0·443
Full-fat milk and milk products†	−0·005	–0·076, 0·065	−0·012	–0·063, 0·024
Whole grain bread and BF cereals‡	0·252***	0·102, 0·381	0·454***	0·192, 0·681
Non-whole grain bread and BF cereal‡	0·312***	0·183, 0·470	0·368***	0·274, 0·553
Fruits	0·271***	0·054, 0·577	0·204***	0·081, 0·614
Vegetables	0·306***	0·211, 0·660	0·423***	0·176. 0·592
Legumes	0·681***	0·355, 1·101	0·543***	0·233, 0·717
Red meat	0·385***	0·182, 0·741	0·431***	0·223, 0·714
White meat and poultry	0·660***	0·221, 0·860	0·448***	0·191, 0·587
Fish and seafood	0·518***	0·310, 0·770	0·582***	0·233, 0·725
Salty snacks	0·072	0·022, 0·183	0·168**	0·072, 0·317
Sweet snacks	0·203***	0·095, 0·451	0·375***	0·123, 0·516
Soft drinks (with sugar)	0·423***	0·215, 0·887	0·558***	0·331, 0·927
Juices (with sugar)	0·501***	0·303, 0·795	0·268***	0·115, 0·443
Water	0·487***	0·250, 0·760	0·387***	0·177, 0·801

*P < 0·05.

** *P* < 0·01.

*** *P* < 0·001 (indicate significance); *β*: Regression coefficient. All analyses were adjusted for age, country, parental sex, educational level and BMI of parents and children.

† Cheese was not counted.

‡ BF: breakfast (rice and pasta were not mentioned under grains group in the questionnaire).

### Compliance of food frequency consumption among parents and children

Table 3 illustrates the frequency of the consumption of food and beverages among parents and children compared to the Food-Based Dietary Guidelines in Europe. Both parents and children did not comply with current dietary recommendations of vegetables, grains (excluding pasta and rice), milk and milk products (excluding cheese). More than 70 % of children consumed more than two servings of sweets per week which exceeded the recommended servings/week^(30)^. 70·9 % of parents and 58·0 % of children were not consuming the daily required number of water cups according to the Food-Based Dietary Guidelines^(30)^.

**Table 3:** Frequency of food consumption among parents and children according to Food-Based Dietary Guidelines in Europe

Foods and beverages	Servings	Dietary Guidelines	Parents (%)	Children (%)
Milk and milk products (no cheese)	< 2 servings/d	Not met	67·3	64·2
	2–3 servings/d	Met	11·5	13·1
	> 3 servings/d	Exceed	21·2	22·7
Grains (bread and BF cereals only)*	< 3 servings/d	Not met	88·3	86·4
	3–5 servings/d	Met	7·40	5·80
	> 5 servings/d	Exceed	4·30	7·80
Fruits	< 2 servings/d	Not met	64·4	61·7
	2–3 servings/d	Met	28·1	30·0
	> 3 servings/d	Exceed	7·50	8·30
Vegetables	< 3 servings/d	Not met	90·4	96·0
	3–4 servings/d	Met	6·50	2·90
	> 4 servings/d	Exceed	3·10	1·10
Legumes	< 2 servings/week	Not met	20·6	30·8
	2–3 servings/week	Met	21·4	23·2
	> 3 servings/week	Exceed	58·0	46·0
Red meat	< 2 servings/week	Not met	16·0	25·7
	2–3 servings/week	Met	30·2	34·2
	> 3 servings/week	Exceed	53·8	40·1
White meat and poultry	< 2 servings/week	Not met	19·0	30·0
	2–3 servings/week	Met	34·8	36·3
	> 3 servings/week	Exceed	46·2	33·7
Fish and seafood	< 2 servings/week	Not met	48·4	62·5
	2–3 servings/week	Met	33·1	26·3
	> 3 servings/week	Exceed	18·5	11·2
Salty snacks	< 1 servings/week	Recommended	22·9	35·0
	1–2 servings/week	Accepted	29·2	36·2
	> 2 servings/week	Exceed	47·9	28·8
Sweet snacks	< 1 servings/week	Recommended	23·0	5·00
	1–2 servings/week	Accepted	29·1	23·0
	> 2 servings/week	Exceed	47·9	72·0
Soft drinks (with added sugar)†	–	N/A	–	–
Juices (with added sugar)†	–	N/A	–	–
Water	< 6 cups/d‡	Not met	70·9	58·0
	6–8 cups/d	Met	20·5	20·4
	> 8 cups/d	Preferred	8·60	21·6

N/A: not applicable.

* BF: breakfast (rice and pasta were not mentioned under grains group in the questionnaire).

† Mentioned in the dietary guidelines as grams of sugar, not as portions or servings of products.

‡ According to the dietary guidelines, the measurement of cups differs between parents (250 ml) and children (150 ml).

### Feel4Diabetes HDS of parents

A higher score indicated higher or more frequent consumption, except for red meat, salty snacks, sugary drinks and sweet snacks where higher scores indicated lower consumption.

The mean total score was 46·6 ± 12·1 among mothers and 43·1 ± 11·2 among fathers (*P* = 0·01). Score value was generally low for vegetables and high for salty snacks and breakfast. In general, the scores were higher among mothers compared to fathers in the red meat group (6·1 ± 3·6 *v*. 4·0 ± 3·8), but almost similar in the salty snacks category (mothers: 5·1 ± 1·3; fathers: 5·0 ± 1·5), milk and milk products (mothers: 3·7 ± 2·1; fathers: 3·7 ± 2·5), oil and fat (mothers: 3·5 ± 2·4; fathers: 3·5 ± 2·2) and sweet snacks (mothers: 3·6 ± 2·0; fathers: 3·7 ± 2·0) (data not shown).

### Association between total parental Healthy Diet Score and children’s food consumption

Table 4 shows the association between parental DQ determined by the HDS and children’s consumption of various food groups. Parental’ HDS was positively associated with girls’ intake of milk and milk products (*β* = 0·152, *P* < 0·01), whole grains (*β* = 0·215, *P* < 0·001), fruits (*β* = 0·134, *P* < 0·05), vegetables (*β* = 0·234, *P* < 0·001) and water (*β* = 0·111, *P* < 0·05) and inversely associated with their intake of salty snacks (*β* = –0·186, *P* < 0·01), sweet snacks (*β* = –0·135, *P* < 0·05) and soft drinks (*β* = –0·202, *P* < 0·001). Among boys, the HDS of parents showed a significant positive association with boys’ intake of full-fat milk and milk products (*β* = 0·173, *P* < 0·01), whole grains (*β* = 0·123, *P* < 0·05), fruits (*β* = 0·233, *P* < 0·001) and vegetables (*β* = –0·177, *P* < 0·01), but a negative association with their intake of legumes (*β* = –0·177, *P* < 0·05), red meat (*β* = –0·206, *P* < 0·001) and salty snacks (*β* = –0·143, *P* < 0·05).

**Table 4:** Association between health diet score of parents and children’s intake from different food groups

	Children’s food consumption				
		Boys		Girls	
Parental healthy diet score	(Food groups)	*B*	95 % CI	*β*	95 % CI
	Low-fat milk and milk products†	0·007	0·002, 0·081	0·152**	0·025, 0·304
	Full-fat milk and milk products†	0·173**	0·065, 0·321	0·124*	0·077, 0·298
	Whole grain bread and BF cereals‡	0·123*	0·077, 0·344	0·215***	0·101, 0·366
	Non-whole grain bread and BF cereal‡	−0·078	–0·143, 0·002	−0·108	–0·201, –0·033
	Fruits	0·233***	0·110, 0·522	0·134*	0·096, 0·301
	Vegetables	0·177**	0·078, 0·290	0·234***	0·102, 0·385
	Legumes	−0·177*	–0·224, –0·052	−0·075	–0·110, 0·026
	Red meat	−0·206***	–0·315, –0·044	−0·101	–0·255, –0·011
	White meat and poultry	−0·033	–0·122, 0·077	0·093	0·065, 0·189
	Fish and seafood	0·044	0·011, 0·089	0·104	0·086, 0·255
	Salty snacks	−0·143*	–0·269, –0·034	−0·186**	–0·239, –0·061
	Sweet snacks	0·014	0·008, 0·098	−0·135*	–0·288, –0·076
	Soft drinks (with sugar)	−0·105	–0·198, –0·084	−0·202***	–0·301, –0·095
	Juices (with sugar)	−0·113	–0·210, –0·044	−0·066	–0·133, 0·015
	Water	0·101	0·055, 0·271	0·111*	0·077, 0·263

* *P* < 0·05.

** *P* < 0·01.

*** *P* < 0·001 (indicate significance); *β*: Standardised Regression coefficient. All analyses were adjusted for age, country, parental sex, educational level and BMI of parents and children.

† Cheese was not counted.

‡ BF: breakfast (rice and pasta were not mentioned under grains group in the questionnaire).

### Association between food consumptions of mothers and children

As shown in Table 5, mothers’ consumption of most food groups was significantly associated with children’s intake among both boys and girls. Mothers’ intake of ‘Full-fat milk and milk products’ was not associated with children’s intake from the same group among both boys and girls. The mothers’ intake of ‘salty snack’ intake did not show any significant association with boys’ intake from the same food group, but it did with that of girls: [(*β* = 0·072, *P* > 0·05) *v*. (*β* = 0·135, *P* < 0·01)].

**Table 5:** Association between dietary intake of mothers and corresponding intake of the same food groups in their children

	Children’s food consumption			
Mothers’ dietary intake (Food groups)	Boys		Girls	
	*β*	95 % CI	*β*	95 % CI
Low-fat milk and milk products†	0·251***	0·091, 0·420	0·341***	0·111, 0·443
Full-fat milk and milk products†	0·013	0·008, 0·067	−0·007	–0·058, 0·023
Whole grain bread and BF cereals‡	0·268***	0·102, 0·344	0·467***	0·211, 0·685
Non-whole grain bread and BF cereal‡	0·288***	0·092, 0·441	0·360***	0·133, 0·540
Fruits	0·271***	0·100, 0·521	0·231***	0·098, 0·422
Vegetables	0·304***	0·185, 0·606	0·442***	0·212, 0·770
Legumes	0·677***	0·324, 1·021	0·560***	0·230, 0·886
Red meat	0·425***	0·202, 0·709	0·417***	0·135, 0·665
White meat and poultry	0·674***	0·287, 0·923	0·474***	0·155, 0·865
Fish and seafood	0·682***	0·388, 1·098	0·492***	0·237, 0·668
Salty snacks	0·072	0·022, 0·132	0·135**	0·092, 0·302
Sweet snacks	0·203**	0·098, 0·440	0·348***	0·112, 0·477
Soft drinks (with sugar)	0·409***	0·165, 0·831	0·540***	0·377, 0·759
Juices (with sugar)	0·561***	0·339, 0·856	0·277***	0·104, 0·411
Water	0·459***	0·250, 0·766	0·368***	0·109, 0·588

*P < 0·05.

** *P* < 0·01.

*** *P* < 0·001 (indicate significance); *β*: Regression coefficient. All analyses were adjusted for age, country, educational level and BMI of mothers and children.

† Cheese was not counted.

‡ BF: breakfast (rice and pasta were not mentioned under grains group in the questionnaire).

### Association between Healthy Diet Score of mothers and children’s dietary intake

Table 6 illustrates the association between the DQ of mothers using HDS and children’s consumption of various food groups. Maternal’ HDS was positively associated with girls’ intake of milk and milk products (*β* = 0·170, *P* < 0·01), whole grains (*β* = 0·245, *P* < 0·001), fruits (*β* = 0·221, *P* < 0·001), vegetables (*β* = 0·238, *P* < 0·001), white meat and poultry (*β* = 0·140, *P* < 0·05), fish and seafood (*β* = 0·182, *P* < 0·01) and inversely associated with their intake of salty snacks (*β* = –0·160. *P* < 0·01), sweet snacks (*β* = –0·127, *P* < 0·05) and soft drinks (*β* = –0·185, *P* < 0·01). The HDS of mothers showed a significant positive association with boys’ intake of full-fat milk and milk products (*β* = 0·202, *P* < 0·01), whole grains (*β* = 0·135, *P* < 0·05), fruits (*β* = 0·231, *P* < 0·001) and vegetables (*β* = 0·175, *P* < 0·01), but a negative association with their intake of legumes (*β* = –0·169, *P* < 0·01), red meat (*β* = –0·248, *P* < 0·001) and salty snacks (*β* = –0·172, *P* < 0·05).

**Table 6:** Association between Healthy Diet Score of mothers and children’s intake from different food

		Children’s food consumption			
		Boys		Girls	
Mothers’ Healthy Diet Score	(Food groups)	*β*	95 % CI	*β*	95 % CI
	Low-fat milk and milk products†	0·016	0·008, 0·049	0·170**	0·034, 0·256
	Full-fat milk and milk products†	0·202**	0·089, 0·422	0·118*	0·087, 0·321
	Whole grain bread and BF cereals‡	0·135*	0·087, 0·344	0·245***	0·102, 0·443
	Non-whole grain bread and BF cereal‡	−0·071	–0·120, 0·003	−0·089	–0·141, 0·024
	Fruits	0·231***	0·103, 0·465	0·221***	0·087, 0·520
	Vegetables	0·175**	0·076, 0·324	0·238***	0·054, 0·419
	Legumes	−0·169**	–0·223, –0·052	−0·037	–0·131, 0·022
	Red meat	−0·248***	–0·331, –0·096	−0·043	–0·114, 0·018
	White meat and poultry	−0·037	–0·101, 0·065	0·140*	0·086, 0·310
	Fish and seafood	0·060	0·022, 0·089	0·182**	0·064, 0·376
	Salty snacks	−0·172*	–0·266, –0·077	−0·160**	–0·225, –0·032
	Sweet snacks	−0·018	–0·097, 0·056	−0·127*	–0·214, –0·044
	Soft drinks (with sugar)	−0·100	–0·201, –0·044	−0·185**	–0·290, –0·057
	Juices (with sugar)	−0·119	–0·255, –0·031	−0·059	–0·122, 0·016
	Water	0·109	0·075, 0·287	0·082	0·043, 0·185

* *P* < 0·05.

** *P* < 0·01.

*** *P* < 0·001 (indicate significance); *β*: standardised regression coefficient. All analyses were adjusted for age, country, educational level and BMI of mothers and children.

† Cheese was not counted.

‡ BF: breakfast (rice and pasta were not mentioned under grains group in the questionnaire).

## Discussion

The present study found that parental food consumption and DQ were significantly associated with children’s consumption of selected food items among boys and girls in families at high risk of T2D. Among the food items, those more associated were FV, grains, milk and milk products. In addition, most parents and children from families at increased risk for T2D showed under-consumption of healthy foods when compared to the European Dietary Guidelines. All these results were found independently of education level, parental sex, age, country and BMI of both parents and children.

In line with the findings of previous systematic reviews on the association between parental and children’s intake^(32,33)^, our results found that parental consumption from FV, legumes, milk and milk products, red meat, poultry, grains, sweets, soft drinks, juices, water, fish and seafood was positively associated with children’s intake from the same food groups. These results suggest that parental dietary intake is strongly linked to children’s food consumption and eating behaviours. Children tend to follow their parents’ diets as seen in a nationally representative data of 1230 parents and children collected by the US Department of Agriculture since parents are considered as role models and food providers^(34)^. Likewise, results from a recent large study across six European countries on 2967 parent–child dyads indicated that children’s dietary intake was strongly associated with the home availability of 100 % fruit juice, also parental role modelling of fruit intake was associated with increased fruit consumption of children^(35)^. Additionally, in the present study, the mean intake of parents and their children were found to be nearly similar in some food groups like fruits, whole grains, milk and milk products. This could be due to the fact that the questionnaire on children’s food intake was completed by parents, which could differ from children’s report of their own diet^(23)^. Moreover, parents may have found it difficult to estimate an average daily consumption of children, particularly the food items that are usually distributed throughout the day in different meals and might be difficult to properly quantify.

Our results found that the majority of parents and their children from families at high risk of T2D did not meet the daily recommendations for FV. Our findings were consistent with those observed by Gerritsen *et al.* ^(36)^ which aimed to compare the children’s intake of FV to the guidelines and generate sustainable actions within a local community to improve children’s FV intake in New Zealand, indicating that children’s FV intake is below than the recommended amount. Among the possible explanations of these results for children is that they get affected by their parents’ dietary intake through role modelling and feeding practices, and thus, children’s FV consumption can be related to their parental FV consumption and the availability of FV at home^(37)^. In addition, children might refuse the consumption of FV because they dislike their taste especially vegetables^(37)^. Moreover, the low FV consumption among parents and children could be also related to the higher prices of healthy foods relative to unhealthy foods, besides the low levels of nutritional knowledge and awareness of parents^(36,37)^.

This study showed that more than 60 % of families at high risk of T2D were not consuming the recommended servings of grains. On the contrary, in a previous study of 1526 preschooler children that aimed to assess the diet of young children attending daycare in the Netherlands, the majority of children was found to meet or exceed the daily recommended intake of grains especially from the refined grains^(38)^. The difference in these results could be due to different tools being used to assess the children’s dietary intake (i.e. FFQ in our study *v*. 2-day food consumption records). Moreover, the FFQ used in our study did not include ‘rice’, ‘pasta’ and other ‘dough products’ under the grains group, but focussed only on bread and breakfast cereals; therefore, the consumption of parents and their children from these food groups might be underestimated in our study.

As shown in our study, the majority of children exceeded the suggested servings of sweets. These results were consistent with previous findings of a cross-sectional study that examined the probability of obesity with higher sweets and sugar intakes in a national representative sample of 781 children and 384 adolescents in Greece, indicating that most of the participants exceeded the recommended intake of sweets and sugar-sweetened beverages^(39)^. Similar results were also observed in a cross-sectional study of 109 children in Ontario, in which 80 % of children had intakes of free sugar greater than the recommended intake^(40)^. In support of this, recent systematic review evaluating the world dietary sugar intake trends in children and adolescents reported that the sugar intakes as a percentage of total energy are the highest for children and adolescents, and despite some reductions in sugar intake observed in a few individual studies, overall intakes of sugars remain above recommendations^(41)^. These results could be explained by the fact that children usually tend to have positive responses to sweets compared to other items with neutral tastes^(42)^. Also, children’s acceptance/refusal of foods and beverages (i.e. sweets and sugar-sweetened beverages) is related to whether they have been repeatedly exposed to them or not during infancy and young ages^(43)^. Food preferences are thought to peak between the age of 2 and 6 years old, so this can shape the child’s DQ later^(43)^.

Additionally, in accordance with previous studies^(44,45)^, in our sample of parents and children, almost half of them did not meet the recommended servings of fish and seafood. The possible explanation of our results could be that parents who do not like fish and seafood themselves may never buy, prepare or offer them to their children^(45)^. Besides, low consumption of fish and seafood among children could be a result of food neophobia and fear from ingesting its bones, its strong aroma and rubbery texture^(45)^. This could also be related to the dietary cultures in the participating countries as the consumption of fish and seafood was found to be higher in fishing areas^(46)^.

The DQ was also considered in this study, in which mothers showed a higher DQ, measured with the HDS, than fathers. The same results were found in a previous randomised clinical trial aimed to compare adults’ DQ scores between seven research centres in Europe, which showed that women tend to have higher DQ than men in Europe using Healthy Eating Index^(47)^, and this could be explained by better nutrition knowledge and awareness in women compared to men^(48)^. Indeed, mothers were found to be the most important source for their children in terms of food consumption and dietary habits through teaching, role modelling and nutritional knowledge^(49)^. Moreover, in our analysis, it has been found that parental gender acts as a moderator in the relationship between parents’ and children’s food consumption. However, it is noteworthy that the majority of the participants in our study were mothers (74·6 %).

Our research demonstrated significant associations between parental DQ and children’s food consumption. Previous studies on the effect of parental DQ on children’s dietary intake demonstrated significant associations between parental DQ and children’s food consumption^(22,23)^, which was indeed confirmed by our results among both boys and girls. Similarly, a large cross-sectional multinational sample of 5185 European families that investigated parental influences on preschool children’s healthy and unhealthy snacking indicated that healthier food choices made by parents were associated with greater child healthy snack intake^(19)^. In depth, our research showed a significant inverse association between parental DQ and the intake of sweets and soft drinks only among girls in families at high risk of T2D. The possible reason of these results could be due to the fact that boys tend to consume more sugar than girls in all age groups^(50)^. Besides, boys’ food preferences and food choices are influenced mainly by taste, whereas girls are usually influenced by how healthy foods are than how they taste^(50)^.

In our sample, both parental and mother’s DQ were significantly and positively associated with boys’ and girls’ intake of FV, whole grains and inversely associated with their snack’s consumption. Although a direct relationship cannot be measured due to the cross-sectional nature of this study, we can assume based on our findings, that improving parental DQ has the potential to positively change children’s food consumptions. These associations are in line with the findings of Davison *et al.* ^(23)^, which aimed to investigate the relationship between parental DQ and child dietary patterns in New Zealand, stated that better parental DQ using the Diet Quality Index (DQI) was associated with children’s lower intake of snacks. But on the contrary, they found no significant association with the children’s consumption of FV. This difference in the results could be due to the use of different tools to identify the quality of a diet (i.e. using HDS in our study *v*. DQI).

There are some limitations of the present study that need to be accounted for. First, children’s data were based on parental report, and this can be considered as a bias, but this weakness is very hard to overcome when studying food intake. Also, some of the children were only in first grades; therefore, it was not possible to get self-reported food intake data from them. Second, the cross-sectional nature of this study does not allow for causality inferences. Moreover, the FFQ is not able to adequately determine absolute food intakes compared to other methods (i.e. 24-hour dietary recall). The FFQ focussed more on breakfast cereal and bread, but other products under the carbohydrates group were not listed (i.e. pasta and rice). Also, cheese was not counted under the group of milk and milk products which may affect the participants’ estimated intake. In addition, a potential correlated bias could be created as the food component scoring of HDS was done according to the fourteen diet-related questions in the Feel4Diabetes questionnaire which is the same tool used to estimate food intake in this study. Furthermore, the response rate contributed to the limitations of this research. Although several strategies were used to improve participation, the low response rate (59·1 %) limits generalisability beyond the study sample. Finally, generalisability of the results is limited to a very specific group of the population, namely members of families with an increased risk on T2D. On the other hand, important strengths of this study included the use of a large data set from six European countries with cultural dietary diversity. Additionally, the anthropometric measurements were obtained by well-trained researchers through using highly validated and standardised procedures to ensure and increase accuracy. Furthermore, to the best of our knowledge, this is the first study to examine the association between parental food consumption, DQ and children’s food consumption in Europe especially among population at high risk of developing T2D.

In conclusion, the present study found that parent’s food consumption of most food groups was associated with the food intake of their children in families at high risk of T2D. Moreover, significant associations were also found between parental DQ and children’s healthy food consumption in boys and girls. However, in families at risk for developing T2D, the food intake of both parents and their children still requires greater emphasis to meet the dietary recommendations. Parents function as role models, who set the rules for their children’s food intake and dietary habits. Therefore, targeting parental food consumption and DQ could be an important strategy to limit the unhealthy eating habits of children and thereby prevent T2D and childhood obesity. More in-depth studies and lifestyle interventions that include both parents and children are therefore recommended for future research.

## Supplementary material

For supplementary material accompanying this paper visit https://doi.org/10.1017/S1368980022002245. click here to view supplementary material
