# Supplementary material for: Parental food consumption and diet quality and its association with children’s food consumption in families at high risk of type 2 diabetes: the Feel4Diabetes-study
Source: Public Health Nutr. 2022 Oct 11;25(12):3344–55. doi: 10.1017/S1368980022002245 (PMC9991723; doi:10.1017/S1368980022002245)
Supplement: Supplementary file 1 [file S1368980022002245sup001.docx]

**Acknowledgements:**

**Members of the Feel4Diabetes-study Group:**

**Coordinator:** Yannis Manios; **Steering Committee:** Yannis Manios, Greet Cardon, Jaana Lindström, Peter Schwarz, Konstantinos Makrilakis, Lieven Annemans, Winne Ko; **Harokopio University (Greece):** Yannis Manios, Kalliopi Karatzi, Odysseas Androutsos, George Moschonis, Spyridon Kanellakis, Christina Mavrogianni, Konstantina Tsoutsoulopoulou, Christina Katsarou, Eva Karaglani, Irini Qira, Efstathios Skoufas, Konstantina Maragkopoulou, Antigone Tsiafitsa, Irini Sotiropoulou, Michalis Tsolakos, Effie Argyri, Mary Nikolaou, Eleni-Anna Vampouli, Christina Filippou, Kyriaki Apergi, Amalia Filippou, Gatsiou Katerina, Efstratios Dimitriadis**; Finnish Institute for Health and Welfare (Finland):** Jaana Lindström, Tiina Laatikainen, Katja Wikström, Jemina Kivelä, Päivi Valve, Esko Levälahti, Eeva Virtanen, Tiina Pennanen, Seija Olli, Karoliina Nelimarkka; **Ghent University (Belgium),** Department of Movement and Sports Sciences: Greet Cardon, Vicky Van Stappen, Nele Huys, Department of Public Health: Lieven Annemans, Ruben Willems, Department of Endocrinology and Metabolic Diseases: Samyah Shadid; **Technische Universität Dresden (Germany):** Peter Schwarz, Patrick Timpel; **University of Athens (Greece):** Konstantinos Makrilakis, Stavros Liatis, George Dafoulas, Christina-Paulina Lambrinou, Angeliki Giannopoulou; **International Diabetes Federation European Region (Belgium):** Winne Ko, Ernest Karuranga; **Universidad De Zaragoza (Spain):** Luis Moreno, Fernando Civeira, Gloria Bueno, Pilar De Miguel-Etayo, Esther Mª Gonzalez-Gil, María L. Miguel-Berges, Natalia Giménez-Legarre; Paloma Flores-Barrantes, Aleli M. Ayala-Marín, Miguel Seral-Cortés, Lucia Baila-Rueda, Ana Cenarro, Estíbaliz Jarauta, Rocío Mateo-Gallego; **Medical University of Varna (Bulgaria):** Violeta Iotova, Tsvetalina Tankova, Natalia Usheva, Kaloyan Tsochev, Nevena Chakarova, Sonya Galcheva, Rumyana Dimova, Yana Bocheva, Zhaneta Radkova, Vanya Marinova, Yuliya Bazdarska, Tanya Stefanova; **University of Debrecen (Hungary):** Imre Rurik, Timea Ungvari, Zoltán Jancsó, Anna Nánási, László Kolozsvári, Csilla Semánova, Éva Bíró, Emese Antal, Sándorné Radó; **Extensive Life Oy (Finland):** Remberto Martinez, Marcos Tong.
